# N2b Reflects the Cognitive Changes in Executive Functioning After Concussion: A Scoping Review

**DOI:** 10.3389/fnhum.2020.601370

**Published:** 2020-12-17

**Authors:** Sophie N. Krokhine, Nathalee P. Ewers, Kiersten I. Mangold, Rober Boshra, Chia-Yu A. Lin, John F. Connolly

**Affiliations:** ^1^Centre for Advanced Research in Experimental and Applied Linguistics (ARiEAL) Research Centre, McMaster University, Hamilton, ON, Canada; ^2^Department of Psychology, Neuroscience and Behaviour, McMaster University, Hamilton, ON, Canada; ^3^Neuroscience Graduate Program, McMaster University, Hamilton, ON, Canada; ^4^School of Biomedical Engineering, McMaster University, Hamilton, ON, Canada

**Keywords:** concussion, event-related potentials, evoked potential, brain injury, sports-related head injury, mild traumatic brain injuries

## Abstract

**Objectives:** The N2b is an event-related potential (ERP) component thought to index higher-order executive function. While the impact of concussion on executive functioning is frequently discussed in the literature, limited research has been done on the role of N2b in evaluating executive functioning in patients with concussion. The aims of this review are to consolidate an understanding of the cognitive functions reflected by the N2b and to account for discrepancies in literature findings regarding the N2b and concussion.

**Methods:** A scoping review was conducted on studies that used the N2b to measure cognitive functioning in healthy control populations, as well as in people with concussions.

**Results:** Sixty-six articles that met inclusion criteria demonstrated that the N2b effectively represents stimulus-response conflict management, response selection, and response inhibition. However, the 19 included articles investigating head injury (using terms such as concussion, mild head injury, and mild traumatic brain injury) found widely varied results: some studies found the amplitude of the N2b to be increased in the concussion group, while others found it to be decreased or unchanged.

**Conclusion:** Based on the available evidence, differences in the amplitude of the N2b have been linked to response selection, conflict, and inhibition deficits in concussion. However, due to large variations in methodology across studies, findings about the directionality of this effect remain inconclusive. The results of this review suggest that future research should be conducted with greater standardization and consistency.

## Introduction

An event-related potential (ERP) is a temporally sensitive, high-resolution trace of electroencephalography (EEG) activity measured over a specific interval of time usually several hundred milliseconds and elicited by a specific group of stimuli or cognitive tasks (Picton et al., 2000; Patel and Azzam, 2005). As such, they are often considered indices of perceptual processes and cognitive functions. Various paradigms, such as the oddball and go/no-go task, are commonly used to elicit ERPs associated with stimulus discrimination, conflict management, attention and memory (Patel and Azzam, 2005). Several ERP components have been studied widely, including: the N1 and P2 (early components that reflect stimulus and feature detection), the P300 (reflecting target selection, memory, and attentional orienting), the error-related negativity or ERN (representing the detection and processing of performance errors), and the N2 (representing focused attention, stimulus discrimination, stimulus-response conflict resolution and response inhibition) (Courchesne et al., 1975; Ritter et al., 1979; Heinze et al., 1990; Eimer, 1997; Larson et al., 2016). It should be noted that a component is capable of reflecting quite different cognitive functions by virtue of the eliciting stimulus paradigm. The P300 response is an excellent example of this phenomenon.

An ERP group of particular focus in this scoping review is N2—which is a negativity resulting from a deviation of prevailing stimulus context that is typically evoked between 180 and 325 ms (Patel and Azzam, 2005). Components within a certain group can be further classified based on their specific scalp topographies and associated cognitive functions; for example, the N2 elicited by attention to rare visual targets, reflecting attentional focusing and stimulus categorization, is maximal over posterior scalp while the N2 elicited by the no-go paradigm, reflecting response inhibition, is maximal over anterior scalp (Debener et al., 2005; Patel and Azzam, 2005; Folstein and Van Petten, 2008; Moore et al., 2015).

The N2 group includes several sub-components such as the N2a (more commonly known as the mismatch negativity), which represents pre-attentive stimulus processing in a manner that has been conceptualized in terms of predictive coding (Friston, 2005; Garrido et al., 2009); the N2c, representing stimulus discrimination and response priming; the N2b, discussed in detail below; the N2p, implicated in target detection and global spatial processing; and the N2pc, implicated in visual search (Näätänen and Picton, 1986; Patel and Azzam, 2005; Folstein and Van Petten, 2008; Van Beek et al., 2015). However, due to the lack of consistency in subcomponent classification, components in this group are often referred to as the general “N2” or by their specific latency, such as, “N270” (Connolly et al., 1995; Helenius et al., 1999; Cui et al., 2000; Kong et al., 2000; Wang et al., 2003, 2004; Bartholow et al., 2005; Patel and Azzam, 2005; Azizian et al., 2006; Folstein and Van Petten, 2008; Luck, 2014). The complexity of N2 is further deepened because it consists of subcomponents originating in different cortical areas, namely in the supplementary motor cortex, left angular gyrus and anterior cingulate cortex (ACC; see Kropotov and Ponomarev, 2009). This review specifically investigates the subcomponent known as the N2b. The N2b, which is generated from ACC (Crottaz-Herbette and Menon, 2006), is thought to primarily reflect higher-order “executive functions” requiring conscious attention (Patel and Azzam, 2005; Downes et al., 2017). N2b is believed to index cognitive processes including response selection and inhibition, stimulus-response conflict adaptation, emotional control, and stimulus discrimination (Courchesne et al., 1975; Czigler et al., 1996; Lange et al., 1998; Smid et al., 1999; Senkowski and Herrmann, 2002; Koivisto and Revonsuo, 2003; Wang et al., 2003; Czigler and Balázs, 2005; Knyazev et al., 2008; Broglio et al., 2009; Mäki-Marttunen et al., 2015). Several studies discussed in the present review utilized paradigms that elicit the N2b while referring to the component with the more general term known as N2. For the purposes of this review, the components in question are to be referred to as the N2b.

Electrophysiology and ERPs have a long history of clinical applications (Chiappa and Ropper, 1982; Regan, 1989), one of which is investigating the effects of concussion. A concussion is a mild traumatic brain injury (mTBI) that is commonly caused by falls and accidents often incurred during contact sports, and results in no observable structural or anatomical changes (McCrory et al., 2017; Panwar et al., 2020). Observable symptoms of concussion, such as dizziness and increased sensitivity to light and sound, typically occur only for seven to 10 days post-injury, although ~20–25% of patients experience symptoms beyond that period in what is termed post-concussion syndrome, or PCS (McCrory et al., 2017). Several studies have identified cognitive and executive functioning deficits, as well as psychiatric disorders such as post-traumatic stress disorder (PTSD) and depression, persisting years post-concussion (Broglio et al., 2009; Gosselin et al., 2012; Martini et al., 2017; Ruiter et al., 2019; Cunningham et al., 2020). However, many others have reported no significant changes in attention, executive functioning or information processing following symptom resolution as evaluated by neuropsychological testing (Potter et al., 2001, 2002; Segalowitz et al., 2001; Guskiewicz et al., 2002).

Standard neuropsychological tests as well as computerized administration of cognitive tests including the Immediate Post-Concussion Assessment and Cognitive Testing (ImPACT) are typically used to measure cognitive and emotional control among people with concussion, yet lack the sensitivity to measure the more subtle manifestations of cognition including executive functioning deficits after symptom resolution (Broglio et al., 2009; Ledwidge and Molfese, 2016; Hudac et al., 2018; Olson et al., 2018). Executive functioning skills, including time management, task switching, sustained attention and working memory, are vital for career and interpersonal success yet can be challenging to accurately measure (Elliott, 2003; Van Beek et al., 2015; Downes et al., 2017). Therefore, more accurate and sensitive measures of chronic cognitive deficits in the post-acute stage of recovery are needed, and ERPs are a promising approach to achieve this objective (Broglio et al., 2009; Ledwidge and Molfese, 2016; Olson et al., 2018).

History of concussion has been associated with reductions in amplitude of the ERN and a reduced and/or delayed P300; however, findings regarding the N2b have been inconclusive (Moore et al., 2015; Ledwidge and Molfese, 2016). While extensive research has been done regarding the N2b, no systematic or scoping review has been conducted to date examining changes in N2b amplitude and latency caused by concussion history, and their relationship to any cognitive implications of concussion.

The objectives of this review are: (1) to identify N2b characteristics and how they differ according to the conditions that elicit the N2b; (2) to consolidate understanding of the N2b's involvement in cognitive functions such as response selection and inhibition, and (3) to identify potential uses of the N2b for measuring cognitive impairment following concussion.

## Materials and Methods

Scoping reviews are conducted to consolidate existing knowledge on a certain topic, identify gaps in the current knowledge, and use these gaps to generate suggestions for future research (Arksey and O'Malley, 2005; Levac et al., 2010). The scoping review typically includes five major steps: (1) Identifying the research question; (2) Identifying relevant studies; (3) Selecting studies for inclusion; (4) Charting the data; and (5) Collating, summarizing, and reporting the results.

### Identifying the Research Question

The research questions addressed in this paper are as follows: (1) What is the N2b and is it a single component? (2) Which cognitive functions are reflected by the N2b and which paradigms are typically used to measure these functions? (3) How do concussions and subconcussive impacts alter the amplitude and latency of the N2b, and how does this reflect apparent cognitive changes?

### Identifying Relevant Studies

The scoping review aimed to identify a comprehensive set of articles that addressed N2b. Literature searches were conducted in July 2019 on the PubMed, EMBASE, PsycINFO, and Web of Science databases for articles investigating the N2b, or general “N2” not further categorized, and concussion (or mTBI). Hand searches were later performed from the reference list of Folstein and Van Petten (2008), an influential review in the field.

### Study Selection

**Figure 1:**
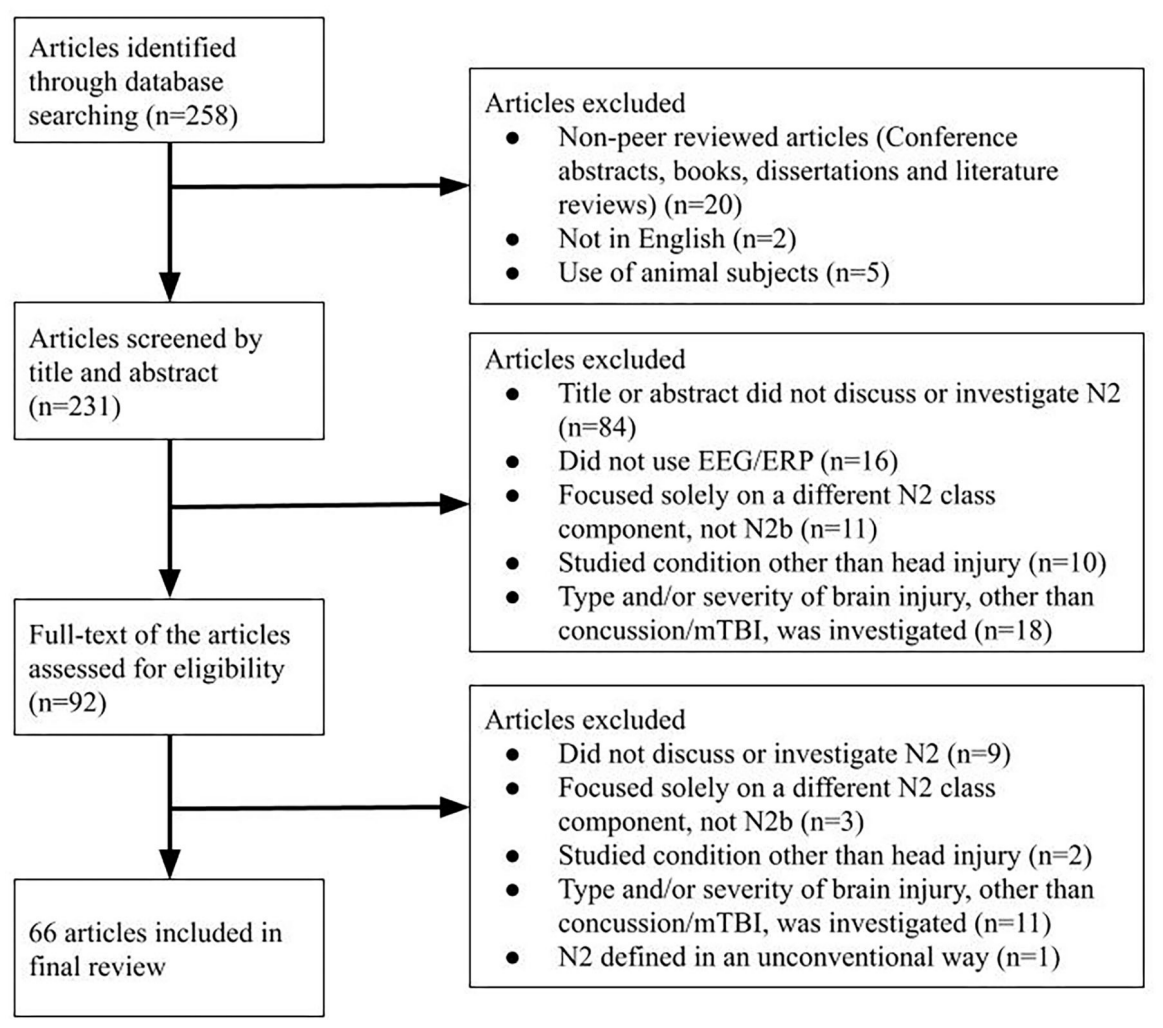
An overview of the selection procedure, with articles included and excluded at each step.

Decisions about inclusion and exclusion of particular articles were made by two reviewers independently (SK and KM), and a third reviewer (NE) made the final decision in the case of disagreement. Inclusion criteria were: primary or secondary focus on the N2 group of ERP components, publication in a peer-reviewed journal in the English language, and usage of human subjects (see Figure 1). Exclusion criteria were publication in a language other than English and usage of animal subjects (Figure 1). Because the N2 had not been separated into sub-components (such as the N2b, N2a, and N2pc) until the late 1980s, studies investigating a general “N2” or “N” followed by a number around 200 (e.g., “N270”) were also included. Studies that specifically investigated the N2b subcomponent were included, while studies that investigated one or more other N2 subcomponents specifically without the N2b were excluded. For the initial screening, the title and abstract of identified articles were reviewed. At this stage, articles were excluded if their title and abstract did not reference the N2 component, cognitive functions of interest such as conflict monitoring and response inhibition, or paradigms such as the oddball task or the Eriksen flanker task. To ensure the comprehensiveness of the search, level of evidence and other methodological limitations were not considered as part of the exclusion criteria.

### Charting the Data

Articles that met the inclusion criteria were examined in detail with an annotated bibliography. They were further categorized based on the population examined, paradigm(s) used, and study objectives to uncover the common themes.

### Collating, Summarizing, and Reporting the Results

The results were organized to target the review's research questions, targeting paradigms, study designs, sample sizes, and participant populations.

## Results

Two hundred and fifty-eight articles were identified from the database searches and reviewed bibliographies, of which 66 were included in the final review (see Supplementary Table 1; Figure 1). Due to the recency of N2 subcomponent classification, the vast majority of included studies referred to the general class of N2 components; only six studied the N2b subcomponent (i.e., Czigler et al., 1996; Lange et al., 1998; Smid et al., 1999; Senkowski and Herrmann, 2002; Czigler and Balázs, 2005; Van Beek et al., 2015). Eight of the included N2 papers specifically investigated concussion (i.e., Broglio et al., 2009; Gosselin et al., 2012; Moore et al., 2014, 2015, 2016; Ledwidge and Molfese, 2016; Hudac et al., 2018; Olson et al., 2018). Eleven studies investigated head injury under a different term: mild head injury (MHI) (i.e., Solbakk et al., 1999; Reinvang et al., 2000; Potter et al., 2001, 2002; Segalowitz et al., 2001), mild traumatic brain injury (mTBI) (i.e., Sivák et al., 2008; Mäki-Marttunen et al., 2015; Van Beek et al., 2015; Zhao et al., 2018; Drapeau et al., 2019), or unspecified head injury (i.e., Andelinović et al., 2015). Results regarding the N2b in healthy subjects and those with concussion are grouped hereafter. Due to the inconsistent use of terminology surrounding concussion, articles using terms such as mTBI or mild head injury are included in this review as noted above. The authors' original use of terms is to be preserved in the following Results and Discussion sections as much as possible.

The N2b is a frontocentral negativity generally peaking between 245 and 290 ms following stimulus onset (Lange et al., 1998; Smid et al., 1999; Senkowski and Herrmann, 2002; Czigler and Balázs, 2005); a wider range between 200 and 400 ms was also reported by Czigler et al. (1996). The N2b is localized to frontal brain regions, specifically the ACC which is responsible for stimulus-response conflict monitoring and resolution. The ERN is also generated in the ACC; thus, the ERN and N2b likely reflect similar conflict and error monitoring processes (Van Veen and Carter, 2002; Nieuwenhuis et al., 2003; Yeung et al., 2004).

Findings regarding the effect of concussion on N2b amplitude and latency have so far been inconclusive. One would assume that the N2b amplitude and/or latency would be altered in individuals with a history of concussion; however, studies have been split on the direction of the effect (Potter et al., 2001; Broglio et al., 2009; Moore et al., 2015; Ledwidge and Molfese, 2016; Olson et al., 2018). One major source of this discrepancy is the different classes of paradigm used to elicit N2b in concussion, including oddball tasks, flanker tasks, go/no-go tasks, n-back working memory tasks, stop-signal tasks, switch tasks, arithmetic tasks, and Stroop tasks. Results corresponding to each paradigm are presented in the following paragraphs. A summary of the included N2 studies involving healthy controls is presented first followed by the findings specific to the concussion population in each of the paradigm sections.

### Oddball Task

One commonly used paradigm is the oddball task, in which subjects are presented with a standard (frequently occurring) and one or more deviant (infrequently occurring) auditory or visual stimuli (Patel and Azzam, 2005; Folstein and Van Petten, 2008; Luck, 2014). Eight articles included in the review examined the N2/N2b using a variant of the oddball task (Breton et al., 1988; Reinvang et al., 2000; Potter et al., 2001; Segalowitz et al., 2001; Sivák et al., 2008; Broglio et al., 2009; Moore et al., 2014; Ledwidge and Molfese, 2016), three in sports concussion specifically (Broglio et al., 2009; Moore et al., 2014; Ledwidge and Molfese, 2016) and four in other forms of head injury (Reinvang et al., 2000; Potter et al., 2001; Segalowitz et al., 2001; Sivák et al., 2008). In this paradigm, subjects are required to respond behaviorally or count deviant stimuli (“targets”) against a stream of standard stimuli (Picton et al., 2000; Patel and Azzam, 2005; Folstein and Van Petten, 2008; Luck, 2014). There are studies using otherwise similar protocols with background standard stimuli and different types of deviants (e.g., involving a change in stimulus duration or intensity compared to the standard). The standard appears more frequently and is typically a tone or image with fixed parameters. Some categories of deviant stimuli are classified as targets which require a response, and others are classified as non-targets which should be ignored. Subjects are asked to produce a different response or withhold all responses to standard stimuli and deviant stimuli that are not targets (Simson et al., 1977; Harter and Guido, 1980; Breton et al., 1988; Reinvang et al., 2000; Ledwidge and Molfese, 2016). An N2 modulation is generally seen in response to deviant stimuli. The size and latency of this modulation is affected by a deviant's status as a target, the total number of targets, and the difficulty of discriminating it from standard stimuli such that it is generally greater for targets than non-targets (Breton et al., 1988; Daffner et al., 2000; Ledwidge and Molfese, 2016). Since slightly different parameters are used in different studies, observed N2 effects differ from study to study (this applies to other paradigms as well). For an example of the time course and topography of the N2b in an oddball paradigm, see Figure 1 on page 116 of Ruiter et al. (2019). In some cases, oddball paradigms also include novel stimuli that differ from immediate context, such as a digit that differs from previously presented digits, or long-term context, such as an unfamiliar geometric figure in a context of triangles in different orientations or an environmental sound (dog bark) in a context to tonal stimuli [Courchesne et al., 1975; Daffner et al., 2000; Suwazono et al., 2000; Segalowitz et al., 2001; Broglio et al., 2009; also see Blain-Moraes et al. (2016) and Mah and Connolly (2018)].

Several studies included in this section used a paradigm very similar to the standard oddball, although the authors did not use the term “oddball” in the articles (i.e., Courchesne et al., 1975; Simson et al., 1977; Ritter et al., 1979; Lange et al., 1998; Daffner et al., 2000; Suwazono et al., 2000). In the study by Lange et al. (1998), subjects switched between responding to different stimulus attributes (location and color). Their task included a location selection condition, in which subjects were expected to respond to stimuli in either the left or right hemifield regardless of their color; a color selection condition, in which they responded to red or blue stimuli regardless of location; and a conjunction condition, in which they responded only to stimuli of one color presented in the attended hemifield.

The oddball N2 is typically used to index stimulus discrimination, response to novel stimuli, and allocation of attentional resources (Ritter et al., 1979; Harter and Guido, 1980; Suwazono et al., 2000; Broglio et al., 2009; Moore et al., 2015). Using an oddball paradigm, Broglio et al. (2009) investigated the influence of concussion on ERP correlates of attention in college-aged athletes with an average of 3.4 years since their most recent concussion. Subjects (athletes aged 18 to 25 playing a wide variety of sports) were divided into two experimental groups: those that had never sustained a concussion (“0” group; *n* = 44) and those that had sustained one or more concussions (“1+” group; *n* = 46). A three-stimulus visual oddball task was used. The concussion history group was found to have significantly smaller N2 and P300 amplitudes, which were believed to reflect deficits in working memory and attention. No significant group differences in latency were found. In contrast, Ledwidge and Molfese (2016) discovered a larger N2 amplitude in collegiate football players who had sustained at least one concussion (with their last concussion an average of 4 years prior; *n* = 22) compared to football players without a history of concussion (*n* = 22), using an auditory oddball task with only one type of deviant. Potter et al. (2001) administered a three-stimulus auditory oddball task to subjects with (*n* = 24) and without (*n* = 24) a history of minor head injury (MHI). The average age of both the MHI and control groups was 27. The injuries of the MHI group were sustained an average of 16.5 months prior to testing and were inflicted by a variety of causes, including rugby, martial arts, motor vehicle accidents, falls and muggings. No significant differences in ERP amplitudes or latencies were observed. Sivák et al. (2008) administered a two-stimulus auditory oddball task to subjects with a concussion history (here referred to as mTBI; *n* = 31) and controls (*n* = 31). Injury was caused by sports impacts, vehicle accidents, falls or fights and time since injury was not reported. No between-group ERP differences were observed. Of those “pseudo-oddball” studies, only Andelinović et al. (2015) investigated subjects with head injury. They observed greater N2 latency for non-targets in the head-injured group as compared to healthy controls.

### Flanker Task

The Eriksen flanker task is a paradigm in which a central visual stimulus is surrounded or “flanked” by distracting stimuli (Eriksen and Eriksen, 1974; Folstein and Van Petten, 2008). The original task developed by Eriksen and Eriksen (1974) used letters as stimuli, but any type of visual stimuli may be used as long as the target and flanker stimuli are distinguishable (Luck, 2014). Different studies have used letters, shapes, or even cartoon fish as stimuli (Heil and Hennighausen, 2000; Yeung et al., 2004; Bartholow et al., 2005; Clayson and Larson, 2012; Moore et al., 2015; Larson et al., 2016; Olson et al., 2018). For example, Kopp et al. (1996b) used a flanker task in which healthy subjects (*n* = 18) were asked to respond based on the direction of the central arrow, and response accuracy and ERPs were measured simultaneously. There were three conditions: congruent (where the flanker stimuli were arrows pointing in the same direction as the central), incongruent (where the flanker arrows were pointing in the opposite direction), and neutral (where the central arrow was flanked by squares that had no indication of direction). Among healthy subjects, the amplitude of the “flanker N2” has typically been found to be largest in the incongruent condition, suggesting that it represents the process of resolving conflict between the different responses primed by the central and flanking stimuli (Kopp et al., 1996b; Van Veen and Carter, 2002; Yeung et al., 2004; Bartholow et al., 2005). For an example of the time course and topography of the N2 in congruent and incongruent conditions of the flanker task, see Figure 11 on page 944 of Yeung et al. (2004).

Olson et al. (2018) administered a modified Eriksen flanker task to college-aged athletes with (*n* = 25) and without (*n* = 22) a history of concussion. The mean ages of both groups were 21. Concussions were incurred by accidents related to sports such as football, soccer and basketball, an average of 2.5 years prior to testing. A non-significant trend of increased N2 amplitude in the concussed athletes was observed. This finding was believed to reflect a greater need for neural resources to compensate for subject-based inefficient conflict monitoring processes, and an overactive performance-monitoring system. Moore et al. (2015) conducted a study on 8-10-year-old children with and without a history of concussion (*n* = 16; control, *n* = 16) using a modified flanker task. Concussions were incurred during sports and recreation activities an average of 2.1 years prior to testing. Cartoon fish were used as stimuli, and subjects were asked to press a button with either their left or right thumb based on the direction of the central fish, and response accuracy and ERPs were measured simultaneously. Flanker stimuli were either congruent (pointing in the same direction) or incongruent (pointing in the opposite direction) to the central fish. To further the investigation of response conflict, the study included both a compatible and incompatible stimulus-response condition. In the compatible condition, the responding hand was compatible with the direction of the central fish (i.e., if the fish was pointing left, subjects were asked to press with their left hand, and vice versa). In the incompatible condition, the responding hand did not correspond with the direction of the central fish (i.e., if the fish was pointing left, subjects were asked to press with their right hand). In the incompatible incongruent condition, subjects had the largest N2 amplitude and lowest accuracy, reflecting increased paradigm-based stimulus-response conflict. In the compatible congruent condition, subjects had the smallest N2 amplitude and greatest behavioral accuracy. The concussion group displayed increased N2 amplitudes in the incompatible condition, and increased N2 latencies in both conditions.

### Go/No-Go Task

The go/no-go task is another commonly used paradigm that tends to elicit the N2b or N2 (see Folstein and Van Petten, 2008). In this task, subjects are asked to respond to a “go” stimulus and withhold this prepotent response when presented with a “no-go” stimulus (Harter and Guido, 1980; Luck, 2014). Similar to the oddball task, this paradigm has been employed in both the visual and the auditory modality (Pfefferbaum et al., 1985; Jodo and Kayama, 1992; Czigler et al., 1996; Falkenstein et al., 1999; Fox et al., 2000; Nieuwenhuis et al., 2004; Dockree et al., 2005). Emotional stimuli, such as happy and fearful faces, have been used in some cases (Mäki-Marttunen et al., 2015). Typically, this task has been used to measure the subject's ability to inhibit the prepotent response (Jodo and Kayama, 1992; Kopp et al., 1996a; Falkenstein et al., 1999; Schmitt et al., 2001a,b) or manage the conflict between the preferred response and the presentation of the no-go stimulus (Moore et al., 2016). Probability manipulations of go and no-go stimuli have been seen to affect the amount of response conflict, and thus amplitudes of N2 (Czigler et al., 1996). See Figure 4 on page 593 of Dockree et al. (2005) for the time course and topography of the N2 in a go/no-go task.

Bruin and Wijers (2002) used a visual go/no-go task (with single-letter stimuli) to measure response inhibition in healthy adults. Subjects were instructed to respond by lifting their left or right index finger when a “go” stimulus was presented, and to withhold this response to a no-go stimulus. Different conditions existed in which the probability of the no-go stimulus was set to 25, 50, and 75%. It was found that a large N2 was elicited in response to no-go stimuli (“the no-go N2”), and its amplitude was increased with decreased probability of the no-go stimulus. Similarly, Nieuwenhuis et al. (2004) administered a go/no-go task in both the visual and auditory modalities. Subjects were expected to respond to, or withhold a response to, the letter “F” depending on the presence of one of two context letters: “T” (which looked similar but sounded different) and “S” (which looked different but sounded similar). When “T” was used as a context letter, a larger N2 was observed in the visual than auditory modality, reflecting increased conflict in the visual modality. In contrast, when “S” was used as a context letter, the N2 in the auditory modality was slightly larger. The authors concluded that the amplitude of the no-go N2 is affected by the degree of perceptual similarity between go and no-go stimuli. As part of the study mentioned in the Flanker Task findings above, Moore et al. (2016) conducted a visual go/no-go task to measure long-term effects of concussion in 8–10-year-old (*n* = 15 and 15 demographically matched controls), in which go and no-go stimuli were cartoon lions and tigers. In the concussion group, increased N2 latency was observed reflecting a deficit in motor inhibition processes. Similar results were found in the switch, but not the n-back, paradigm employed in their study. Mäki-Marttunen et al. (2015) performed an emotional go/no-go task on subjects with mTBI (*n* = 27) and controls (*n* = 17). Neutral stimuli (flowers) and threatening stimuli (spiders) were used, in different conditions, as go, no-go, and distractor (irrelevant) stimuli. Both groups displayed increased N2 amplitudes to threatening stimuli, regardless of their status as go, no-go and distractor. In the mTBI group, go N2 latency was reduced for threatening stimuli.

### N-Back Working Memory Task

The n-back working memory task is a paradigm in which subjects are presented with a series of stimuli, one at a time, and are asked to determine whether the current stimulus is the same as the one presented a certain number of trials ago (Moore et al., 2016; Hudac et al., 2018). Within the same study mentioned above, Moore et al. (2016) also employed 0-, 1-, and 2- back tasks in addition to the switch task and the go/no-go task to assess concussed children (*n* = 15 and 15 demographically matched controls). In the 0-back condition, subjects were asked to respond with their right thumb only if they saw a cross and with their left thumb for all other shapes. In the 1-back condition, subjects were asked to respond with their right thumb if the shape was the same one presented on the previous trial, and in the 2-back condition subjects were asked to respond with their right thumb if the shape was the same one presented two trials ago. No between-group ERP differences were observed. Hudac et al. (2018) used a 2-back working memory task among college football players with concussion (*n* = 17; controls, *n* = 19). Subjects were expected to determine whether a letter stimulus matched the one from two trials ago. Reduced N2b amplitude and latency was observed for the concussion group. Gosselin et al. (2012) also used a visual working memory task in which adults with (*n* = 44) and without (*n* = 40) a history of mTBI were presented with a series of four images, then asked whether a fifth image matched any one of the four previously presented. While the concussion group displayed reduced behavioral performance and P300 amplitude, no changes in N2 amplitude or latency were observed. See Figure 1 on page 5 of Gosselin et al. (2012) for an example of time course and topography of the N2 wave in this paradigm.

### Stop-Signal Task

The stop-signal task is a paradigm similar to the go/no-go task in which subjects are expected to continuously respond to a series of “go” stimuli and cease responding when a “stop” stimulus is introduced shortly after the “go” stimulus (Kok et al., 2004; Ramautar et al., 2004, 2006; Schmajuk et al., 2006; Knyazev et al., 2008; Luck, 2014). For example, Ramautar et al. (2004) conducted a study in which subjects were presented with visual go stimuli and a visual stop signal of varying probability (20 or 50%). N2 and P300 latencies were observed to be increased in the 50% probability condition, and P300 amplitude was increased in the 20% condition. Ramautar et al. (2006) later determined that the N2 amplitude was larger on unsuccessful stop trials, reflecting a possible error monitoring function. See Figure 4 on page 242 of Ramautar et al. (2004) for the time course of the N2 wave in a stop-signal task. Knyazev et al. (2008) conducted an auditory stop-signal task, in which subjects had to press the left button on their keyboard after hearing a high-pitched sound (2,000 Hz) and the right button after hearing a low-pitched sound (1,000 Hz). When a click was presented after the tone, subjects were expected to inhibit their response. ERP measures on successful and unsuccessful stop trials were compared, and both N2 and P3 latencies were greater on unsuccessful trials. The authors suggested that this difference reflects a failure of attention and conflict monitoring processes, rather than response inhibition. No studies using the stop-signal paradigm in concussion were found.

### Switch Task

Another paradigm that has been used to measure N2b is the task-switching paradigm or “switch task,” which measures subjects' ability to transfer attention from one task to another, as task switching is thought to be another cognitive skill impaired in concussion (Monsell, 2003; Moore et al., 2014, 2016). For example, Moore et al. (2014) studied the cognitive impacts of concussion in young adults (concussion group, *n* = 19; control, *n* = 21; mean age = 21) using 3-stimulus oddball, flanker, and numerical switch task paradigms. Subjects had sustained concussion as children or adolescents, an average of 7.1 years prior. In the switch task subjects were first presented with trials of two homogeneous tasks, then a heterogeneous task. In the first homogeneous task, a number was presented visually within a box with a dashed outline. Subjects were asked to identify whether the number was odd or even. In the second task, numbers were presented in a solid box and subjects were asked to identify whether the number was smaller or greater than 5. In the heterogeneous task, subjects were presented with numbers in either a dashed or solid box and asked to switch between responding to the different rule sets. In the switch condition of this task, the box “switched” from dashed to solid or vice versa and subjects were asked to respond using a different rule set. In the non-switch condition, subjects used the same rule set twice in a row. The global switch condition represented the difference between homogeneous and heterogeneous tasks; while the local switch condition represented the differences between switching rule sets and repeating the same rule set (“switch” and “non-switch” trials). In the heterogeneous condition, subjects in both groups exhibited longer reaction times and reduced behavioral accuracy. Group differences in N2 amplitude and latency varied with condition; the concussion group displayed increased N2 amplitudes for the heterogeneous conditions of the local and global switch tasks. In the local switch task, this effect was only visible across non-switch trials. Between-group latency differences were observed in both the global and local switch tasks: for the global switch task, subjects in the concussion group exhibited shorter N2 latencies across both switch and non-switch trials; while for the local switch task, subjects exhibited shorter latencies in switch trials and longer latencies in non-switch trials. See Figure 3 on page 31 of Moore et al. (2014) for the time course of the N2 wave in a switch task.

### Arithmetic Task

Van Beek et al. (2015) assigned 7–12-year-old children who had sustained an mTBI 6–30 days prior to testing (*n* = 16) and healthy control children (*n* = 16) to perform single-digit addition problems. Some problems had large sums (>10) while others had smaller sums (<10). The authors specifically investigated the N2b component, not the general N2. In both groups, behavioral accuracy was lower for the problems with larger sums. Twenty-five percent of the mTBI group displayed significantly worse behavioral performance than the comparison group, but no group differences in N1, N2b or P2 amplitudes and latencies were observed.

### Stroop Task

In the Stroop task, subjects are presented with words naming different colors (e.g., “blue,” “black,” “red”) printed in a color congruent or incongruent to the name, and are asked to respond to either the name or color (Folstein and Van Petten, 2008; Luck, 2014). For example, in the congruent condition the word “blue” would be printed in blue, while in the incongruent condition it might be printed in red or yellow. The paradigm has been used to study conflict management, because the incongruent condition requires subjects to suppress the response suggested by the opposite attribute. Potter et al. (2002) administered a Stroop task to adults with (*n* = 24) and without (*n* = 24) a history of mild head injury (MHI). The words “red,” “green,” “blue,” and “yellow” were displayed both on printed cards (card-based task) and a computer screen (computer-based task) in congruent and incongruent ink colors, and subjects were instructed to state the name of the ink color. In both the computerized and physical modalities, reaction times were significantly longer in the incongruent condition, suggesting a greater allocation of attentional resources required for conflict resolution. Subjects in the MHI group displayed reduced behavioral accuracy, but no changes in N2 amplitude or latency, in both modalities. In the card-based task, subjects in the MHI group exhibited greater reaction times; while in the computer-based task, they exhibited a larger late negativity in the 350–450 ms latency range, but not within the defined N2 window of 180–325 ms (Patel and Azzam, 2005). See Figure 3 on page 834 of Potter et al. (2002) for the time courses of ERPs in a Stroop task.

### Non-standard Discrimination Tasks

Non-standard stimulus discrimination tasks, which do not fit into any of the aforementioned categories, have been used in several ERP studies. In the choice reaction time task (CRT), subjects are expected to produce one response to target stimuli and a different response to non-targets or novel stimuli (Ritter et al., 1982; Eimer, 1997; Kong et al., 2000; Senkowski and Herrmann, 2002; Azizian et al., 2006; Zhao et al., 2018). In other paradigms, subjects respond to matches or mismatches of form, color, or shape between two visual stimuli (Harter and Guido, 1980; Breton et al., 1988; Heinze et al., 1990; Lange et al., 1998; Smid et al., 1999; Cui et al., 2000; Wang et al., 2003, 2004; Czigler and Balázs, 2005). Harter and Guido (1980) used a discrimination task involving horizontal and vertical black-and-white gratings and diffuse light; on each trial, subjects were asked to respond to one relevant stimulus type while ignoring other stimulus types. A negative component around 235 ms (termed “N235a”) was elicited by task relevant stimuli over occipital scalp. N2 amplitude was generally greater for attended stimuli (Heinze et al., 1990; Eimer, 1997). While N2/N2b amplitude was influenced by perceptual similarity, the direction of this effect was unclear. Some studies reported greater amplitude for more similar stimuli (Senkowski and Herrmann, 2002; Azizian et al., 2006) while others reported the opposite (Kong et al., 2000; Wang et al., 2003).

Drapeau et al. (2019) used an emotional discrimination task in which subjects with and without a head injury history were presented with a stream of happy and fearful facial expressions, and expected to respond to rare non-emotional stimuli (butterflies). Larger N2 amplitudes were observed to the fearful facial expressions, and there were no between-group differences. Okita et al. (1985) used a working memory discrimination task, in which subjects attended to one diagonal of a display and searched for targets amid “dot masks.” A negativity around 200 ms, not specifically termed N2b, was elicited by higher memory load in the attended diagonals. In the “change-blindness” task used by Koivisto and Revonsuo (2003) subjects were expected to respond to a change in a stimulus feature, and a greater N2 amplitude was observed on correctly detected change trials. Response decisions have been based on semantic as well as phonological or syntactic features, particularly in studies of language production (Ritter et al., 1983; Schmitt et al., 2000, 2001a,b). Increased N2 latencies have been observed when the go/no-go decision is consistent on phonological rather than semantic information (Schmitt et al., 2000, 2001a,b).

While the majority of these non-standard tasks were in the visual modality, there was one in the auditory modality. Solbakk et al. (1999) administered a dichotic listening task to a group of 15 subjects who had sustained a mild head injury of unspecified cause, and 13 age-matched controls. The MHI group had a mean age of 41 years, and had sustained injury an average of 6.2 years prior to testing. The task had a monaural condition, in which participants attended to stimuli presented only in one ear, and a dichotic condition, in which they attended to stimuli in one ear while ignoring the other. Decreased N2 amplitudes were seen in the MHI group, both for the monaural condition and the attended ear in the dichotic condition. Reduced N2b amplitude was observed in those subjects with a history of mild head injury.

## Discussion

A wealth of past research has shown that the N2b represents various higher-order “executive functions” including response selection, conflict management, and inhibition. While many of the studies cited in the results referred to the component in question as “N2,” it can be argued that this component can be subclassified as the N2b. While some other subclasses of the N2, such as the N2a (or mismatch negativity), reflect pre-attentive and automatic processing often conceptualized in a predictive coding framework (Friston, 2005; Patel and Azzam, 2005; Folstein and Van Petten, 2008; Garrido et al., 2009; Luck, 2014), the N2b reflects processing modulated by conscious attention to stimuli (Lange et al., 1998; Smid et al., 1999; Senkowski and Herrmann, 2002). Different paradigms measure different cognitive functions associated with the N2b: the N2b elicited by the oddball paradigm typically represents stimulus discrimination and response to novelty (Daffner et al., 2000; Reinvang et al., 2000; Suwazono et al., 2000; Broglio et al., 2009), while the N2b elicited by the flanker and go/no-go tasks mainly represents response conflict management and inhibition (Pfefferbaum et al., 1985; Fox et al., 2000; Heil and Hennighausen, 2000; Schmitt et al., 2001a,b; Yeung et al., 2004; Azizian et al., 2006; Moore et al., 2015). The amplitude of the no-go N2b typically increases with the amount of conflict required to inhibit the prepotent response; conflict can be increased by altering the probabilities of the go- and no-go stimuli, or altering the physical characteristics and thus similarity between the stimuli (Pfefferbaum et al., 1985; Nieuwenhuis et al., 2003, 2004; Clayson and Larson, 2012). Discrepancies in the effect of concussion on the N2b are surprising given that findings regarding other components (such as the P300 and ERN) have been fairly consistent. The vast majority of studies have found the P300 to be reduced after concussion, regardless of whether an oddball, flanker or go/no-go paradigm was used (Broglio et al., 2009; Moore et al., 2014, 2016). Studies investigating the ERN have also found significant increases following concussion (Moore et al., 2015; Olson et al., 2018). Results involving the N2b as it relates to concussion seem to vary strongly across different studies. Overall, the N2b components elicited by each group of paradigms represent disparate cognitive functions that manifest differently in concussion; therefore, standardization of paradigms and consideration of further subclassification of the associated N2b components is essential for future research. Of note, while the studies reported varying results in amplitude and latency of the N2b following concussion, there were no reports of changes in scalp topography post-injury, a factor that should be investigated in future work.

Findings regarding the N2b and concussion have remained fairly inconclusive. Studies have found the N2b amplitude to be increased, decreased, or unchanged following a concussion; there is no prevailing result because there is no prevailing N2b. This lack of consensus is thought to be due to the great variation among studies in methodology and sample characteristics that should be incorporated to further our understanding of brain injury (as suggested by Olson et al., 2018). An assumption of comparability and invariance (e.g., of different age, time-since-injury, and experimental paradigm) is the wrong approach and creates an expectation that is simply not supported by the extant literature (Boshra et al., 2020). Differences in the labeling and classification of components could also contribute to these inconsistencies: some studies reported on the N2b component specifically (Czigler et al., 1996; Lange et al., 1998; Smid et al., 1999; Senkowski and Herrmann, 2002; Czigler and Balázs, 2005; Van Beek et al., 2015) while others referred to it as the general “N2” (Heil and Hennighausen, 2000; Broglio et al., 2009; Gosselin et al., 2012; Moore et al., 2015; Ledwidge and Molfese, 2016; Olson et al., 2018; Zhao et al., 2018) which could be a combination of the multiple currently identified sub-components. In addition to the observed lack of consensus in methodology and terminology in current literature, the number of identified concussion-specific studies under each paradigm is too small for any conclusive summary to be drawn. Studies investigating the general N2 used a wide variety of paradigms (including oddball tasks and n-back working memory tasks), while studies specifically looking at the N2b typically used oddball, go/no-go, or flanker tasks. While the N2b is known to represent response selection and inhibition as measured by go/no-go, Stroop and flanker tasks (Czigler et al., 1996; Lange et al., 1998; Smid et al., 1999; Senkowski and Herrmann, 2002; Czigler and Balázs, 2005; Van Beek et al., 2015), the general N2, which encompasses all the current sub-classifications, almost certainly represents a much broader range of cognitive functions and thus will present differently in different paradigms and experimental manipulations (Patel and Azzam, 2005; Folstein and Van Petten, 2008; Luck, 2014).

The paradigms themselves also differ significantly across studies, introducing another source of variability. In other words, an “oddball” paradigm used by one study may be strikingly different from an “oddball” paradigm used by another study. About half of the reviewed studies were performed in the auditory modality, while the rest were performed in the visual modality—only three included experiments in both (Falkenstein et al., 1999; Nieuwenhuis et al., 2004; Ramautar et al., 2006). As auditory and visual processing occur through different neural pathways, cognitive functioning in one modality may be more sensitive to concussion (Ramautar et al., 2006). There were also between-study differences for the classes of stimuli used (e.g., shapes, letters, numbers, and arrows), time of presentation and inter-stimulus interval, emotional associations of the stimuli, and probability of target or no-go stimuli—variables reported to affect the N2b amplitude (Breton et al., 1988; Falkenstein et al., 1999; Fox et al., 2000; Dockree et al., 2005; Azizian et al., 2006; Schmajuk et al., 2006; Mäki-Marttunen et al., 2015).

Another variable that will affect the consistency and validity of these results is the age of the study subjects. N2 and P300 amplitude have been shown to increase throughout child development but reduce with further aging, representing a general slowing of cognitive processing (Czigler et al., 1996; Czigler and Balázs, 2005; Larson et al., 2016). The studies investigated in this review vary widely across age groups of subjects, from 8-year-old children to adults over 65, so the incorporation of the variance of age and its effect on findings from the literature is important (Czigler et al., 1996; Czigler and Balázs, 2005; Moore et al., 2015, 2016; Ruiter et al., 2020).

Another factor that might affect ERP correlates of cognitive functioning in concussion is the severity of the head injury sustained. Six of the head injury studies reviewed (i.e., Solbakk et al., 1999; Sivák et al., 2008; Gosselin et al., 2012; Van Beek et al., 2015; Zhao et al., 2018; Drapeau et al., 2019) used a standard sports concussion definition similar to that proposed by McCrory et al. (2017), which to some extent improved similarity across sample groups. However, many studies did not select subjects by this definition; some included subjects with more profound symptoms such as unconsciousness and post-traumatic amnesia, and still others provided little or no information about the severity of subjects' head injuries (e.g., Andelinović et al., 2015). It is believed that subjects with more severe head injuries would experience more profound cognitive deficits, leading to more pronounced effects on ERP amplitudes. Many older players self-reported concussions and were not officially diagnosed, so the characteristics of their head injuries are difficult to determine and compare.

Overall, the results of this review suggest that N2b components might best be classified based on the eliciting paradigm. The oddball N2b, which represents selective attention and novelty response, is smaller in subjects with a head-injury history (e.g., Ledwidge and Molfese, 2016; Ruiter et al., 2019). On the other hand, the flanker N2b, which represents response conflict management and inhibition, is reportedly larger (Moore et al., 2015; Olson et al., 2018). If we assume that these differential manifestations of the N2b are robust, then the N2b is not a singular phenomenon but rather a response linked to very different cognitive processes. These processes may be differentially affected by concussion and exhibit distinct differences during acute, post-acute and chronic stages of concussion; and distinct trajectories of recovery or deterioration during the transition from post-acute to chronic stages.

## Conclusions and Future Directions

These discrepancies in the relationship between the N2b component and recovery from concussion highlight three major areas in which future research could be improved: (1) Increasing standardization of methodology; (2) Improving consistency in the terminology used to describe head injuries, and (3) Clarifying further subclassification of the N2b.

To increase standardization of studies, subject variables including age and recency of head injury must be better controlled. This could be accomplished through subject matching, or simply by narrowing the scope of the study to a specific subgroup (such as college-aged athletes). Studies should recruit subjects based on the common definition of sports concussion (e.g., McCrory et al., 2017) and standard protocols for paradigms (such as the oddball task and flanker task) should be developed and utilized. In the guidelines for using human ERPs to study cognition by Picton et al. (2000), specific recording standards and publication criteria were listed in detail. The implementation of such rigorous reporting, if adopted by all, would certainly make comparisons among various studies more achievable.

It is also important to note that the N2b components elicited by various paradigms are associated with different, albeit related, cognitive functions that present differently in concussion. To reduce ambiguity and increase the clarity of results, N2b could be denoted by several sub-components elicited by the various paradigms. The Go/No-Go N2b could be denoted as the “N2b_go/no−go_” sub-component, representing response inhibition. Similarly, the Oddball N2b could be denoted by the “N2b_oddball_,” representing stimulus discrimination and novelty response. The flanker and Stroop N2b could be referred to as the “N2b_conflict_,” which could represent stimulus-response conflict management. Future concussion research would likely focus on the N2b_go/no−go_ and N2b_conflict_, as concussion would have the greatest impact on these “higher-order” executive functions (Olson et al., 2018).

The current study was not without limitations. There is a lack of consistency in the literature in the terminology describing the N2b and concussion. Many articles referred to the N2b as the N2, or N270 and components with these labels may have had slightly different amplitudes, latencies, topographies and neural representations. The decomposition of the N2 as shown in Kropotov and Ponomarev (2009) further highlights the complexity of N2; that is, the three identified neuronal generators (i.e., supplementary motor cortex, left angular gyrus, and ACC) were associated with action suppression, sensory comparison and conflict-monitoring operation, respectively (Kropotov and Ponomarev, 2009). In their review, Folstein and Van Petten (2008) also allude to the challenge in N2 classification in the existing literature. Articles also used different labels for concussion, such as mild head injury and mild traumatic brain injury, which may not have been consistent in terms of injury severity and characteristics.

Future research must be done to solidify these subclassifications, and determine the associated effects of concussion. Studies should include sufficiently large samples of both healthy subjects and those with a history of concussion, and should employ longitudinal and cross-sectional designs whenever possible. The N2bs elicited by different common paradigms could be compared across the same subjects, to further isolate the effect of paradigm characteristics on the N2b component and possibly help develop the aforementioned sub-classifications.

In conclusion, this scoping review describes the current state of knowledge about the N2b component as well as how it has been measured in concussion. Suggestions for improving the quality and consistency of future concussion research are proposed. Understanding the cognitive impacts of concussion and the tools available to measure them is vital to enable individuals who have experienced concussions to access appropriate timely treatment and rehabilitation services.

## Author Contributions

NE and JC contributed to the conception of the study. C-YL contributed to the design of the study. SK organized the database, led the search, and wrote the first draft of the manuscript. SK, KM, and NE reviewed the studies. NE, KM, RB, C-YL, and JC wrote sections of the manuscript. All authors contributed to manuscript revision, read, and approved the submitted version.

## Conflict of Interest

The authors declare that the research was conducted in the absence of any commercial or financial relationships that could be construed as a potential conflict of interest.
