# Supplementary material for: N2b Reflects the Cognitive Changes in Executive Functioning After Concussion: A Scoping Review
Source: Front Hum Neurosci. 2020 Dec 17;14:601370. doi: 10.3389/fnhum.2020.601370 (PMC7793768; doi:10.3389/fnhum.2020.601370)
Supplement: Supplementary file 1 [file Table_1.pdf]

Supplementary Material

Supplementary Table 1. Detailed summary of articles included in the N2b and concussion scoping review.

Study	Sample Characteristics and Paradigms Used	Main Objectives	Key Relevant Findings
HEALTHY STUDY SUBJECTS			
Azizian et al., 2006	17 healthy subjects, aged 18-28; Visual go/no-go and choice reaction-time task, manipulation of stimulus probability with similar and dissimilar nontargets	To determine how N2 related to magnitude of stimulus-response conflict	Increased N2 for similar nontargets as opposed to targets and dissimilar nontargets, although P3 largest for targets
Bartholow et al., 2005	42 healthy subjects aged 21-30; traditional flanker task with letters as stimuli; 3 probability conditions: 50% congruent/50% incongruent (expect-neutral), 80% congruent/20% incongruent (expect-compatible) and 20% congruent/80% incongruent (expect-incompatible)	To examine how the N2 and correct response negativity (CRN) relate to response conflict and are affected by stimulus probability	Increased N2 amplitude on incompatible trials (in which flanking stimuli differed from targets); Increased N2 also seen for more probable stimuli; CRN and ERN greater for incompatible trials
Breton et al., 1988	9 healthy subjects, age not reported; Visual oddball task and choice reaction-time/stimulus matching (including 1-target, 25-target and matching pairs target conditions)	To determine the effect of stimulus probability and number of possible targets on the N2	Increased N2 amplitude in 25-target compared to 1-target, smaller amplitude in matching pairs condition.

Bruin and Wijers, 2002	12 healthy subjects, aged 19-28, mean age=21.5; Visual go/no-go task (manual response and silent counting, manipulation of go stimulus probability-25%, 50%, 75%)	To investigate effect of stimulus probability on the N2	Increased amplitude of no-go N2 and P3 with increased go stimulus probability (decreased no-go probability), in both the presence and absence of overt responding; Increased amplitude of go N2 in 50% and 75% go probability conditions, but decreased amplitude of P3 with increasing stimulus probability
Clayson and Larson, 2012	89 healthy subjects, aged 18-22, mean age=21; Flanker task, neuropsychological memory and attention tests (WAIS-III digit span test, Trail Making Test, Controlled Oral Word Association Test-COWAT, Category Fluency Test, Rey Auditory-Verbal Learning Test-RAVLT) administered to investigate relation to ERP results; 4 classes of flanker trials identified: congruent preceded by congruent trial (cC), congruent preceded by incongruent trial (iC), incongruent preceded by congruent (cI) and incongruent preceded by incongruent (iI)	To investigate how N2 modulations reflect conflict adaptation	Increased N2 amplitude on incongruent trials and following congruent trials-conflict adaptation effects (congruent vs incongruent difference effects of amplitude) increased with higher scores on attention/executive functioning measures; Increased reaction times in iI, iC and cI conditions

Courchesne et al., 1975	18 healthy university students (age not reported) were involved in a passive visual experimental task and counting tasks (in which they were asked to count the number 4, and novel and simple/easily recognizable stimuli)	To investigate the effect of task-relevant and task-irrelevant novel stimuli on the P300	Increased N2 amplitude, decreased N2 latency and increased P3 amplitude in response to novels as opposed to simples or 4s; N2 larger for counted than passively viewed 4s, but no effect of task relevance for novels and simples
Cui et al., 2000	14 healthy subjects, aged 21-29; Subjects were presented with two consecutive stereo or plane shapes, and asked whether they were identical; In condition 1, subjects were presented with identical stereo shapes; in condition 2, identical plane shapes; in condition 3, different stereo shapes; and in condition 4, different plane shapes	To investigate the neural mechanisms involved in visual processing of stereo and plane shapes	A negative component (N270) was observed in both difference conditions; it was posteriorly located for stereo shapes and anteriorly located for plane shapes, suggesting that stereo and plane shapes are processed using different neural mechanisms
Czigler and Balász, 2005	Healthy subjects, older group (n=8, age 62-75, mean age=70.5) and younger group (n=8, age 19-23, mean age=21.8); Visual letter matching task with novel stimuli	To determine how novelty processing, indexed by the N2b, is affected by age	Novelty-related N2b and P3b observed solely in younger group, also smaller early components (P1, N1, P2). Indicates age-related difficulties with novelty processing
Czigler et al., 1996	Healthy subjects: older (n=10, age 57-68, mean age=61.4) and younger (n=10, age 18-24, mean age=22.8); Visual go/no-go with stimulus probability manipulation	To determine the effect of age on early stimulus identification processes, and of stimulus probability on the N2b	Decreased amplitude and increased latency of N2b in older subjects, also longer latency of early NA component

Daffner et al., 2000	24 healthy subjects, mean age=21; Visual standard, target and deviant discrimination task (all-simple condition-no novel stimuli, all-novel condition-all novel stimuli, mixed-stimuli condition-standard and target stimuli only were novel); Similar to an oddball task, but the authors did not actually use the word "oddball"	To determine how N2 is related to novelty processing	Increased N2 amplitude in all-novel and mixed-stimulus conditions, compared to all-simple condition, for all stimulus types; In all-novel condition, N2 amplitude greatest for deviants and targets; In mixed-stimuli condition, amplitude greatest for standards
Dockree et al., 2015	14 healthy adult subjects, aged 18-32, mean age=23.86; Visual Go/no-go task, in which subjects were asked to inhibit a response to one digit (e.g. 3) flanked by a predictable sequence of digits (from 1 to 9)	To determine and characterize ERP components related to attention: selection negativity (SN), late positive 1 (LP1) and late positive 2 (LP2)	Increased N2 amplitude seen in no-go trials; It was thought to represent target selection and motor response inhibition processes; Increases in SN, LP1 also seen in predicted-target trials
Eimer, 1997	12 healthy subjects, aged 21-37, mean age=28.8; Visual choice reaction-time task (attended targets and unattended stimuli, attention to form or color for stimuli); Sustained and transient attention conditions	To investigate mechanisms of spatial and non-spatial attention under transient and sustained conditions	Increased N2 amplitude associated with stimulus attention, greater effect for sustained condition; Smaller effect for form than color

Falkenstein et al., 1999	10 healthy subjects, aged 18-33, mean age=24.1; Auditory and visual go/no-go task used, letters F and J as equiprobable stimuli; Auditory and visual stimuli interspersed; F and J alternate as go and no-go stimuli across blocks	To test the inhibition hypothesis for the no-go N2, and examine differences in visual and auditory modality; to investigate relationship of N2 to error-related negativity	Smaller amplitude of the no-go N2 in the auditory modality; Increased no-go N2 amplitude and decreased N2 latency in subjects making fewer commission errors, particularly in visual modality; Lack of modality differences and different scalp topography of the ERN suggests it originates differently from N2
Fox et al., 2000	8 healthy subjects, aged 19-41, mean age=23.1; Visual go/no-go task (both elemental and configural-conjunction of two features- no-go stimuli used)	To determine how N2 reflects response inhibition in the go/no-go task	Increased N2 amplitude observed in no-go trials, N2 larger in elemental than configural trials
Harter and Guido, 1980	6 healthy subjects, age not reported; Unspecified visual discrimination paradigm (black and white gratings-vertical and horizontal orientation-as well as diffuse light used as stimuli)	To investigate the role of N2 components (N235a, N260a) in stimulus selection processes	N235a observed over occipital scalp regions, sensitive to task relevance and grating orientation; N260a sensitive to difference between grating and diffuse light
Heil et al., 2000	18 healthy subjects, age 21-33, mean age=25; Hybrid go/no-go and flanker task	To investigate how N2 reflects response inhibition	Increased N200 amplitude in incompatible go condition and specifically primed no/go condition, suggesting a role in motor inhibition

Heinze et al., 1990	3 experiments were run with different groups of subjects: Experiment 1 (n=12, age 18-29), Experiment 2 (n=7 from the 12 in Experiment 1), Experiment 3 (n=8, 3 from Experiment 1 and 5 new, age not reported); All were visual letter matching tasks with "probe" (standard) stimuli	To investigate how ERP components reflect attention and stimulus selection	Increased N2 amplitude for attended (not unattended) targets as compared to standards; N1 larger for attended targets as well
Jodo and Kamaya, 1992	20 healthy subjects, aged 20-24; Visual go/no-go task with short (high inhibition/HI) and long (low inhibition/LI) response time conditions	To investigate how N2 reflects response inhibition	Increased N2 amplitude on no-go trials, particularly in HI condition; Results support inhibition role of N2
Knyazev et al., 2008)	51 healthy subjects, aged 18-30, mean age=20; Auditory stop-signal task	To investigate the cognitive processes underlying successful and unsuccessful stopping	For Stop trials, N2 and P3 latencies were greater, and P3 amplitude was smaller on unsuccessful trials; For Go trials N2 had smaller latency and P3 had larger amplitude on unsuccessful trials; The authors concluded failed stopping is associated with an uncharacteristically strong response to Go stimuli and weaker response to Stop stimuli; Success or failure to stop is mostly determined by response preparation and attentional control processes
Koivisto and Revonsuo, 2003	12 healthy subjects, aged 21-25; Visual stimulus discrimination (“change-blindness”) task	To determine ERP correlates of change detection and “change blindness”	Increased N2 amplitude in correctly detected change trials

Kok et al., 2004	12 healthy subjects, aged 18-33, mean age=23; Stop signal and choice reaction-time, with square and circle used as stimuli; 50% stop-signal probability, 18 evenly spaced stop-signal delays between 10 to 300ms; Reaction time and delays split into 'early', 'middle' and 'late' bins	To investigate ERP correlates of primary stimulus detection and stop-signal processing, and cognitive differences in successful vs. unsuccessful stopping	Number of unsuccessful stop trials (USSTs) directly related to stop-signal delay; N2/P3 complex observed for both successful and unsuccessful stop trials, but greater amplitude and latency on USSTs. USST N2 has central topography
Kong et al., 2000	14 healthy subjects, aged 21-39 were presented with a series of two numbers, that were either the same (matching) or different (mismatch)	To investigate ERP correlates of semantic mismatch and conflict processes	A negative component (N270) was observed in the mismatch condition, thought to reflect perceptual conflict
Kopp, Mattler, et al., 1996	18 healthy subjects (mean age=33); Visual hybrid go/no-go and flanker task	To investigate ERP correlates of conflict monitoring and response inhibition on specifically and nonspecifically primed go/no-go trials	N2 observed only on incongruent go trials and specifically primed no-go trials
Kopp, Rist et al., 1996	18 healthy subjects, mean age=33; Flanker task with congruent, incongruent and neutral conditions; Arrowheads used as stimuli and squares used as neutral stimuli	To investigate ERP correlates of response inhibition and response priming (when stimuli are partially analyzed thus activating a response)	N2b appeared in all three flanker conditions, amplitude greater for congruent and incongruent than for neutral; N2c in incongruent only, P3 larger in neutral and incongruent; Significant effects of facilitation (neutral-congruent comparison) and interference (neutral-incongruent)
Lange et al., 1998	8 healthy subjects, aged 21-27, mean age=24; Visual selective attention task	To investigate ERP correlates of feature/color selection	N2b observed in both color, location, and conjunction selection- centrally maximal topography

Larson et al., 2016	Two groups of subjects: Older (n=65, age 55-85, mean=69.03) and younger (n=94, age 18-30, mean=21.02); Large sample sizes are notable; Eriksen flanker task	To investigate the effect of age on conflict adaptation and performance monitoring	Decreased N2 amplitude in older as opposed to younger adults, increased reaction time and error rate in older adults
Nieuwenhuis et al., 2003	12 undergraduate students, aged 18-24, mean age=20.9; Visual go/no-go task with manipulation of no-go stimulus probability (20%, 50%, 80%)	To investigate N2 as a measure of stimulus-response conflict and how it is affected by stimulus probability	Increased N2 amplitude for the rare go and no-go stimuli; N2 and ERN both localized to the anterior cingulate cortex, suggesting role in conflict not inhibition
Nieuwenhuis et al., 2004	12 young adults, mean age=23.5; Visual and auditory go/no-go task (go and no-go stimulus similarity manipulated in both modalities- go letter "F", visually similar letter "T" and similar sounding letter "S").	To investigate the effect of stimulus similarity in the no-go N2 and determine why other studies did not find an N2 in the auditory modality	Increased N2 amplitude in visual modality when visually similar context letter used, increased amplitude in auditory modality when similar sounding context letters used
Okita et al., 1985	2 experiments performed of a visual discrimination task (working memory load, attending to one diagonal and looking for targets, "dot masks" included): Experiment 1 (n=8, age not reported) and Experiment 2 (n=4, age not reported)	To determine the stages of information processing, and ERP correlates of attention and working memory	Negativity around 200ms observed in response to increased memory load when stimuli are attended

Pfefferbaum et al., 1985	Different groups were included: Experiment 1 (n=12, aged 20-29), Experiment 2 (n=8, aged 20-29), and Experiment 3 (n=10, aged 20-29); Subjects completed a go/no-go task, in which the word "PUSH" triggered a Go response and the word "WAIT" was a No-Go signal; In half of the trials, the words were modified with ampersands to make them more difficult to discriminate (&P&U&S&H& and &W&A&I&T&); Vertical and horizontal lines of Os also used as go and no-go stimuli (which could be degraded); In experiment 1, subjects were expected to press a button in response to Go stimuli (PUSH or vertical Os); In experiment 2, subjects pressed a button for go stimuli in one condition and counted them in another; In experiment 3, only the symbols were presented: in one condition they were as before (go=vertical line and no-go=horizontal) and in another they were reversed (go=horizontal and no-go=vertical)	To investigate cognitive differences in responding to semantic vs. symbolic stimuli, to determine ERP results specifically associated with the motor response, and to investigate ERP differences caused by changes in symbol orientation	For all 3 experiments, N2 amplitude was greater for no-go than go stimuli; The topography of P3 was also different (parietal for go and central-parietal for no-go); Across experiments 1 and 2, N2 amplitude and latency were not affected by stimulus type (word vs symbol); In experiment 2, N2 latency was decreased, and the Go/No-Go effect became larger, in the pressing condition; In experiment 3, stimulus orientation had no effect on N2 amplitude/latency. The effect of degrading was different across experiments: In experiment 1, degraded stimuli elicited a larger N2 with no change in latency; P3 amplitude was increased for symbol stimuli only, and latency increased for all but the symbolic No-Go stimulus; In experiment 2, degrading caused no N2 changes although P3 amplitudes for symbols were increased; In experiment 2, degrading had no effect on N2 but prolonged P3 latency

Ramautar et al., 2004	14 healthy subjects, aged 18-23, mean age=20.14; Visual stop-signal and choice reaction time tasks (manipulation of stop-signal delays and probabilities- 20% and 50%)	To determine how N2 and P3 relate to inhibition in the stop-signal task, and how they are affected by stimulus probability	Greater N2 amplitude and latency on unsuccessful stop trials; Greater N2 and P3 latency in the 50% stop-signal probability condition as opposed to the 20% condition
Ramautar et al., 2006	15 healthy subjects, aged 18-24, mean age=21.2; Visual and auditory stop-signal and choice reaction time tasks (50% stop-signal probability only)	To determine the effect of modality (auditory or visual) on N2 and P3 generated by stop-signal task	Decreased N2 and increased P3 amplitude for go trials, increased N2 amplitude for unsuccessful stop trials in visual modality
Ritter et al., 1979	4 healthy subjects, age not reported; Unspecified auditory discrimination task used in which subjects asked to respond to random pitch changes. "Easy" and "Hard" discrimination conditions	To investigate ERP correlates of stimulus discrimination	Increased N2 latency in hard vs. easy condition, N2 latency correlated with P3 latency and reaction time; N2 may represent target selection and its latency is correlated with reaction time
Ritter et al., 1982	11 healthy subjects, age not reported; Visual classification and working memory tasks (task A, discriminating between letters and numbers and task B, between letters that were or were not part of a memory set); Half of stimuli were degraded by Xs	To test the hypothesis that early N_A and N2 reflect sequential stages of stimulus processing	N2 consistently occurring after N_A in both tasks A and B. Degrading stimuli increased peak latency of N_A; Bigger interval between N_A and N2 latency for task B

Ritter et al., 1983	12 healthy subjects, age not reported; Visual stimulus discrimination task (physical vs semantic, probability manipulation); Responding to 20% rare stimuli amid 80% frequent stimuli; In physical condition, responding to change in stimuli-reversal of brackets or angles; In semantic condition, responding to male/female names or animal/non-animal words; Simple reaction-time task-responding to all stimuli	To build on the results of Ritter et al. (1982) and investigate whether N_A and N2 are also generated in semantic discrimination	Increased N2 amplitude associated with stimulus changes and rare stimuli, regardless of their status of targets; N_A and N2 also observed in semantic discrimination, but N2 had different scalp distribution
Schmajuk et al., 2006	11 healthy subjects, mean age=22; Visual stop-signal task (with "stop irrelevant" trials in which subjects ignored stop signal)	To investigate ERP correlates of response inhibition	Increased right frontal N200 observed in successful vs. failed inhibitions; Increased occipito-parietal N200 observed in Stop Relevant vs Stop Irrelevant trials
Schmitt et al., 2000	15 native speakers of German, aged 26-36, mean age=29; Visual go/no-go task (decision contingent on semantic vs phonological information)	To investigate the time course of semantic vs. phonological processing	Increased N200 latency when the go/no-go decision was contingent on phonology as opposed to semantics
Schmitt, Rodriguez-Fornells, et al., 2001	2 experiments with different groups of subjects: Tacit picture naming (n=18, mean age=30) and listening study (n=16, mean age=23); Go/no-go decision consistent on syntax vs semantics	To investigate the time course of semantic vs. phonological processing	Increased N200 latency in the go/no-go=syntax condition as opposed to go/no-go=semantics; N200 NOT correlated with reaction time

Schmitt, Schlitz et al., 2001	Sample size and age not clearly reported; Visual go/no-go task (tacit picture naming; responding hand and no-go consistent on conceptual and syntactical information in different conditions)	To investigate the time course of semantic vs. phonological processing	Decreased N200 latency when go/no-go decision contingent on conceptual information
Senkowski and Herrmann, 2002	9 healthy subjects, mean age=23.4; Visual stimulus discrimination task used with colored circles (easy task-green vs red and hard-light green vs dark green)	To investigate cognitive/ERP differences in easy and hard discrimination tasks	Increased posterior N2b and P3 for hard discrimination task. EEG evoked gamma activity appeared later in hard task
Simson et al., 1977	8 healthy subjects, aged 21-45; Visual and auditory discrimination task similar to oddball, detecting frequent (non-signal) and rare (signal) stimuli	To investigate scalp topography of ERPs associated with rare stimuli	Increased N2 for targets/signals as opposed to non-targets/non-signals; In auditory condition, N2 had central distribution (around the vertex) while in visual condition, it had parietal-occipital distribution
Smid et al., 1999	18 healthy subjects, aged 19-29, mean age=24.5; Visual discrimination task (color, global and local shape)	To investigate ERP correlates of stimulus selection (SN, FSP, N2b) - whether they reflect same or different processes	N2b observed in response to color relevance and global shape, occurred in conjunction with selection negativity (SN) and frontal selection positivity (FSP)

Suwazono et al., 2000	Experiment 1 (n=8, mean age=27), Experiment 2 (n=8, mean age=21.1), Experiment 3 (n=7, mean age=25.1); Visual "detection task", similar to typical oddball task with novel stimuli; target predictability of novels manipulated (100% predictable- Experiment 1, 40%- Experiment 2 and 20%-Experiment 3)	To investigate how N2 was affected by predictability of novel stimuli	Increased amplitude of target N2 in experiments 2 and 3 where the novel stimuli were not fully predictive
Van Veen and Carter, 2002	12 healthy subjects, mean age=23.4; Flanker task (included the typical "response-incongruent" condition and a "stimulus-incongruent" condition where flanking stimuli were different from central but provoked same response)	To determine the time course of conflict-monitoring processes in the anterior cingulate cortex (ACC)	Increased N2 amplitude in response-incongruent condition compared to congruent and stimulus-incongruent conditions, localized to ACC
Wang, Cui et al., 2004	15 healthy subjects, aged 19-30); Visual stimulus discrimination task (comparing one stimulus to another with respect to color and shape); Single mismatches - only color or shape is different, conjunction mismatches - both are different; Attend-color, attend-shape and attend-color and shape conditions included	To determine how N270 and N400 reflect processing of stimulus mismatch	N270 elicited in single mismatch conditions, greater amplitude when task-relevant; N270 and N400 appear sequentially only in conjunction mismatches where both attributes are attended

Wang, Tian, et al., 2003	13 healthy subjects, aged 20-30; Visual stimulus discrimination task (comparing one stimulus to another with respect to color, global shape and local shape)	To investigate the time course of visual conflict monitoring	N270 elicited by completely mismatching stimuli (second stimulus differing from first in all three attributes)
Yeung et al. (2004)	16 healthy subjects, aged 18-23; Flanker task	To investigate ERP correlates of conflict monitoring, providing a model for generation of the error-related negativity (ERN)	Increased N2 latency on incongruent trials, related to increase in reaction time; ERN and N2 have similar scalp topography; N2 peaks before response while ERN peaks after
STUDY SUBJECTS WITH CONCUSSION/HEAD INJURY/MTBI			
Andelinović et al., 2015	15 football players with exposure to headers and 15 control subjects, all aged 16; Unspecified auditory discrimination task similar to oddball	To determine how N2 amplitude and latency is affected by changes in brain function following concussion	Increase in N2 latency of head-injured group in response to non-target stimuli; Increased P3 latency for frequent stimuli
Broglio et al., 2009	46 subjects with mTBI and 44 controls, aged 18-25, mean age=19.7; Visual oddball task with novel stimulus	To investigate effect of concussion on attentional processes, as indicated by the N2	Decreased N2 and P3b amplitude in concussion group, possibly reflecting deficits in attentional orienting and response inhibition
Drapeau et al., 2019	10 subjects with mTBI (age 20-46) and 11 healthy controls (age 28-51); Visual emotional discrimination task (asked to count non-emotional target stimuli)	To investigate changes in attentional and emotional processing following head injury	Increased N2 amplitude for fearful as opposed to neutral and happy expressions, no between-group differences; Only N1 was affected (amplitude reduced) in mTBI group; Suggested that emotional processing remains intact

Gosselin et al., 2012	44 patients with concussion (7.6 ± 8.4 months postinjury) and age and sex-matched controls (n=40), aged 16-60; Visual working memory task used.	To investigate the effect of concussion on working memory processes and related ERP measures	Unchanged N200 amplitude or latency between groups, but decreased N350 and P300 amplitudes
Hudac et al., 2018	36 male college American football athletes, aged 18-23, mean age=20.84; 17 with concussion history (2.94 years since concussion) and 19 healthy control; Standard 2-back visual working memory task	To examine the relationship of N2 to working memory impairments in sports concussion	In concussion group, reduced N2 latency and larger P1 and P3 amplitudes observed; ERP modulations thought to reflect deficits in attentional resource allocation and working memory
Ledwidge and Molfese, 2016	44 collegiate football players with (n=22) and without a history of concussion (n=22), mean age=20, participated in a two-tone auditory oddball task as well as neuropsychological tests (D-KEFS Neuropsychological Battery, Trail-Making Test, WAIS-IV); Athletes in the concussion group were in the post-acute stage of recovery, with an average of 4 years since injury	To determine the effects of sports concussion on attentional processing	Neuropsychological test measures did not differ between the two groups; In the oddball task, the concussion group showed greater N2/P3b amplitude and delayed P3b latency, representing a decrease in attentional processing efficiency and a subsequent increase in resource allocation; These findings support the hypothesis that other brain regions will compensate for an injured region, restoring functionality but requiring more resources to sustain

Mäki-Marttunen et al., 2015	27 subjects with a history of mTBI and 17 controls with previous ankle injury participated in a Go/No-Go visual discrimination task, in which neutral (flower) and threatening (spider) stimuli were used as Go, No-Go or distractor stimuli	To investigate how mTBI affects attention capture by emotional stimuli, whether relevant or irrelevant to the task	In all Go and No-Go conditions, both groups showed an increase in N2/P3 amplitude in response to threatening stimuli, which was larger in the mTBI group; The mTBI group also exhibited an increased Go response speed to threat-related stimuli, suggesting increased allocation of attentional resources to emotionally provoking or threatening stimuli
Moore et al., 2014	19 young adults with previous concussion (7.1 ± 4.0 years from injury) and 21 aged-matched controls, aged 20-29, mean age=21.3; Visual oddball, switch and flanker tasks	To determine long-term effects of concussion on attention and executive function	Increased N2 and reduced P3 amplitude in concussion group during heterogeneous condition of switch task; Decreased N2 latency in switch trials and increased N2 latency in nonswitch trials; No significant N2 results for oddball or flanker tasks
Moore et al., 2015	8-10-year-old children with (n=16) and without (n=16) a history of concussion participated in a visual Eriksen flanker task, with the target stimulus as a cartoon fish; Congruent condition (where flanking stimuli matched target stimulus) and an incongruent condition (where they did not); Frequency of omission errors (not responding due to lapses in attention) was also monitored	To determine how pediatric concussion affects stimulus attention, response preparation and error monitoring	Concussion group made significantly more omission errors, and demonstrated increased N2 amplitude (during the incongruent condition only) and latency (during both the congruent and incongruent conditions); The authors concluded that children with a history of concussion experience greater stimulus-response conflict, which is resolved less quickly

Moore et al., 2016	8-10-year-old children with (n=15) and without (n=15) a history of concussion participated in visual n-back, go/no-go and switch tasks	To investigate ERP correlates of attention, working memory and cognitive control deficits post-concussion	Increased N2 latency and decreased N1 amplitude (significant in switch task and non-significant trend in go/no-go task)
Olson et al., 2018	Adult athletes aged 18-30 (mean age=20 years) with (n=25) and without (n=22) a history of sports concussion completed a modified Eriksen flanker task; Response accuracy and reaction time were measured	To investigate differences in conflict resolution and error monitoring in concussion	Accuracy and reaction time did not significantly differ across groups, most likely due to compensatory mechanisms; Larger (more negative) N2 and ERN amplitudes were observed in the concussion group; This suggests that conflict resolution processes may be impaired in those with a concussion history, requiring greater resource allocation to resolve stimulus-response conflict
Potter et al., 2001	24 subjects with mild head injury (MHI; aged 18-44, mean age=27) and 24 controls (aged 16-50, mean age=27) participated in a 3-stimulus auditory oddball task	To investigate working memory and attention deficits in mild head injury	Target condition N2 deflection shown to be larger in MHI group but not significant; There were no other significant differences in behavioral accuracy and ERP amplitudes or latencies between MHI and control groups, which runs contrary to the findings of many previous studies
Potter et al., 2002	24 subjects with MHI (aged 16-54, mean age=32) and 24 controls (aged 18-46, mean age=31.4) participated in Stroop task (both computer-based and printed card-based)	To investigate ERP correlates of attentional processing in MHI	Increased late negative deflection around 350-450 ms, non-significant N2 increase in MHI group

Reinvang et al., 2000	52 subjects with a head injury history (mean age=32.8) and 27 controls (mean age=29.7) participated in a standard two-stimulus auditory oddball task; There was both an active condition, where subjects were required to press a button in response to a target, and a baseline or neutral condition, where a non-target stimulus was repeatedly presented	To determine the effect of MHI on early attentional and conventional cognitive ERP components (N1, P2, N2, P3)	P2 amplitude was lower in the MHI group for both the neutral and active conditions, while no significant group differences in N1 amplitude or latency were found; The latency of N2 was also slightly slower in the MHI group; This evidence supports the hypothesis that head injury reduces stimulus discrimination ability, leading to nonoptimal distribution of attentional resources
Segalowitz et al., 2001	10 young adults with MHI (aged 19-23, mean age=20.1, average length of time since injury was 6.4 years) and 12 controls (aged 19-32, mean age=20.67) participated in four auditory oddball conditions: pitch deviant, pitch deviant with added novel stimuli, duration deviant, and duration deviant with added distractor task; Subjects also completed standard neuropsychological tests	To investigate whether ERP recording is a more sensitive and accurate method for measuring post-MHI cognitive deficits than neuropsychological tests	While neuropsychological test performance did not differ significantly across the two groups, the MHI group displayed a reduced P300 amplitude; P300 amplitude was inversely related to task difficulty; No changes found in N1, P2 and N2 components; This suggests that recording of ERP components, particularly the P300, is an effective method of measuring persistent cognitive deficits related to head injury

Siválk et al., 2008	31 subjects with mTBI (aged 18-54, mean age=32) and 31 controls (aged 18-52, mean age=29.7); Auditory oddball task used (with MRI)	To investigate ERP correlates of attention and discrimination, relate them to MRI findings in mTBI	Unchanged N2 and P3 amplitudes and latencies in mTBI group; 8 subjects in mTBI group and 7 in control group showed pathologies on MRI; Oddball paradigm could not detect cognitive deficits
Solbakk et al., 1999	15 subjects with MHI (mean age=41, mean time since injury was 6.2 years), 10 subjects with frontal brain injury (mean age=40, mean time since injury was 5.1 years) and 13 aged matched controls; Dichotic listening task (responding to stimuli in one ear while ignoring stimuli in the other ear)	To investigate ERP correlates of attention and stimulus detection in MHI/frontal lesions	Decreased N2 amplitudes in MHI group (compared with frontal and control groups)
Van Beek et al., 2015	16 children recovering from mTBI (mean age=10y8m, between 6-30 days post injury) and 16 healthy controls (mean age=10y9m) participated in a simple addition task; ERP data was simultaneously recorded	To investigate neural abnormalities underlying arithmetic difficulties in children with mTBI	Children with mTBI performed significantly worse on the larger arithmetic problems; No group differences in P1, N1, P2 and N2 but displayed a smaller amplitude of the late positive component, which represents higher-order and attention-dependent processes
Zhao et al. (2018)	Subjects: mTBI (n=20) and age-matched controls (n=20), age 20-55 for both groups. Visual continuous performance task (AX-CPT, including a go/no-go element); Event-related synchronization and desynchronization (ERD/ERS)	To investigate N2 and P3 as correlates of response inhibition and conflict monitoring deficits in mTBI.	Decreased N2 amplitudes in mTBI group (compared to controls) in both Go and No-Go conditions. Decreased P3 amplitude in mTBI for Go condition only. ERS/ERD decreased in certain brain regions at varying times after stimulus onset.
